# Microwave-Assisted Chemical Activation of Caraway Seeds with Potassium Carbonate for Activated Carbon Production: Physicochemical Characterization and Adsorption Study

**DOI:** 10.3390/molecules30183804

**Published:** 2025-09-18

**Authors:** Dorota Paluch, Aleksandra Bazan-Wozniak, Robert Pietrzak

**Affiliations:** Department of Applied Chemistry, Faculty of Chemistry, Adam Mickiewicz University in Poznan, Uniwersytetu Poznanskiego 8, 61-614 Poznan, Poland; dorota.paluch@amu.edu.pl (D.P.); aleksandra.bazan@amu.edu.pl (A.B.-W.)

**Keywords:** activated carbon, caraway seed, microwave-assisted activation, chemical activation, methylene blue, methyl red

## Abstract

This study reports the production of carbon adsorbents via microwave-assisted chemical activation of caraway seeds using potassium carbonate (K_2_CO_3_). Microwave irradiation enables rapid, energy-efficient heating, promoting effective pore development at relatively low activation temperatures (400–600 °C). The resulting carbons were comprehensively characterized in terms of surface area, pore structure, and surface chemistry, and their adsorption performance was evaluated for both cationic (methylene blue) and anionic (methyl red) dyes. The adsorbents exhibited specific surface areas ranging from 25 to 634 m^2^/g, with sorption capacities up to 217 mg/g for methylene blue and 171 mg/g for methyl red. Adsorption kinetics followed a pseudo-second-order model, and isotherm analysis revealed that Langmuir adsorption predominates for methylene blue, while Freundlich adsorption better describes methyl red uptake, reflecting surface heterogeneity. This work demonstrates that caraway seeds are a low-cost, sustainable precursor for producing microwave-activated carbons and provides new insights into the influence of activation temperature and surface chemistry on dye adsorption mechanisms, highlighting the practical potential of these materials for wastewater treatment applications.

## 1. Introduction

Chemical activation is a widely used method for producing activated carbon with high surface area and well-developed porosity. Among various chemical activating agents, potassium carbonate (K_2_CO_3_) has gained attention as a relatively mild yet effective activating agent, especially for biomass-derived precursors. Unlike more aggressive chemicals such as KOH or ZnCl_2_, K_2_CO_3_ offers the advantage of lower corrosivity and safer handling, while still enabling the development of a rich porous structure [1]. During the activation process, the precursor material is first impregnated with an aqueous solution of K_2_CO_3_, typically at a specific impregnation ratio (precursor:activator), followed by thermal treatment at elevated temperatures (usually 400–800 °C) under an inert atmosphere. The decomposition of K_2_CO_3_ at high temperatures generates CO_2_ and K_2_O, which play key roles in creating porosity. CO_2_ contributes to gasification of the carbon matrix, while K_2_O may react with the carbon to form intermediate potassium compounds that facilitate the opening and widening of pores [2,3]. K_2_CO_3_ activation typically favors the development of mesopores and macropores, although a notable amount of micropores can also form depending on the activation temperature and the nature of the precursor. The milder reactivity of K_2_CO_3_ leads to less structural degradation, resulting in activated carbon with a more stable and ordered architecture. Additionally, potassium-based activation promotes the incorporation of basic surface functionalities, which can enhance adsorption of acidic or polar contaminants, such as phenols or dyes [4,5]. Another advantage of using potassium carbonate lies in its environmental compatibility. After activation, residual potassium compounds can be easily removed by washing with dilute acid and water, generating minimal toxic byproducts [6].

Numerous studies have demonstrated that K_2_CO_3_-activated carbon performs efficiently in the removal of contaminants from both aqueous and gaseous media. For instance, activated carbons prepared using K_2_CO_3_ have shown high adsorption capacities for dyes such as methylene blue and heavy metals like Pb^2+^ and Cd^2+^, owing to their high surface area and functionalized pore structures [7]. Thus, K_2_CO_3_ remains a promising agent for environmentally friendly and cost-effective production of activated carbons from various low-cost biomass sources.

Conventional thermal methods rely on heat transfer mechanisms such as conduction, convection, and radiation, which often lead to uneven heating and thermal gradients within particles. This inefficiency has prompted interest in alternative heating technologies, notably microwave-assisted activation. Microwave heating offers several advantages: rapid internal heating, volumetric energy absorption, shorter processing times, and lower energy consumption compared to traditional techniques [8]. Moreover, it enables the production of more uniform adsorbent structures with higher surface area and porosity. Microwave radiation works by converting electromagnetic energy into thermal energy directly within the material, allowing for more homogeneous temperature distribution. This internal heating eliminates the need for external heat sources and reduces the temperature differentials between the core and surface of the particles [9]. As a result, material synthesis under microwave conditions tends to be faster, more energy-efficient, and more controllable. In traditional heating, the outer surface of the particle is heated first, with heat gradually transferring inward. This often causes volatile compounds to migrate outward, potentially leading to structural inconsistencies [10]. Conversely, microwave heating initiates at the core and progresses outward, reversing the thermal gradient. Under pyrolytic conditions, this inward-outward flow of volatiles enhances decomposition efficiency and allows carbonization at lower temperatures [11]. Overall, microwave-assisted activation represents a modern, eco-friendly approach to producing high-performance carbon materials with enhanced porosity and surface functionality [12]. Its advantages make it a compelling alternative to conventional thermal processes, especially in the context of sustainable materials engineering and environmental remediation.

The growing challenges of unsustainable material consumption and ineffective resource management have accelerated the global shift toward circular economy principles, particularly in the area of waste valorization. In industrial settings, the demand for low-cost, abundant raw materials-combined with stringent life cycle assessments-continues to drive the adoption of more sustainable practices and greener chemical processes. This study investigates the synthesis and characterization of activated carbon materials derived from an unconventional biomass feedstock: caraway seeds, a waste by-product of the herbal industry. Chemical activation was carried out using potassium carbonate (K_2_CO_3_), a relatively mild and environmentally friendly activating agent. To improve energy efficiency and heating uniformity, microwave irradiation was utilized as the thermal source. Activation was performed at three distinct temperatures—400 °C, 500 °C, and 600 °C—to systematically examine the effects of thermal conditions on pore structure, surface chemistry, and adsorption performance. This research presents a novel approach to transforming agricultural waste into cost-effective bioadsorbents capable of removing organic dyes from water. The findings contribute to both waste reduction and the advancement of sustainable materials for environmental remediation. Previous studies have demonstrated the effectiveness of microwave-assisted activation for producing porous carbons from agricultural residues such as coconut shells, nutshells, and lignocellulosic by-products [13,14]. Compared to conventional heating, microwave irradiation provides rapid, energy-efficient, and volumetric heating, enabling effective pore development at lower activation temperatures and shorter processing times. Most reports on K_2_CO_3_ activation have focused on these conventional precursors and methods, typically achieving surface areas between ~500 and 1200 m^2^/g and evaluating adsorption mainly for cationic dyes [7,15,16]. However, limited research has been conducted on caraway seeds as a precursor, despite their abundance as an agricultural by-product. Moreover, few studies have systematically compared adsorption of both cationic and anionic dyes under identical conditions. In this work, we address these gaps by producing microwave-activated carbons from caraway seeds, characterizing their structural and surface chemistry features, and evaluating their adsorption performance toward methylene blue and methyl red. This dual-dye approach, combined with kinetic and thermodynamic analysis, provides new insights into adsorption mechanisms and the relationship between activation parameters, surface functionality, and sorption capacity, thereby extending the understanding of microwave-assisted K_2_CO_3_ activation.

## 2. Results and Discussion

**Figure 1:**
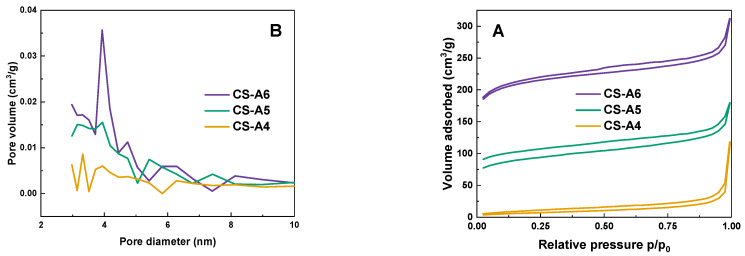
Low-temperature N_2_ adsorption-desorption isotherms (**A**) and pore size distribution (**B**) of activated carbon samples obtained.

Figure 1 presents the low-temperature nitrogen adsorption-desorption isotherms (A) and the corresponding pore size distributions (B) for the obtained activated carbon samples. According to the IUPAC classification system, six types of hysteresis loops are defined, each corresponding to specific pore structures and adsorption mechanisms. All samples exhibited Type IV isotherms with H2-type hysteresis loops, indicative of well-developed mesoporous structures. This loop shape is typically associated with pores that have narrow necks and wider bodies—commonly referred to as ink-bottle-shaped pores [17]. The characteristic feature of this isotherm is the occurrence of capillary condensation in pores with diameters within the mesoporous range [18].

The porous structure of the samples was generated through microwave-assisted chemical activation using K_2_CO_3_. During the activation process, K_2_CO_3_ reacts with the carbon precursor at elevated temperatures, producing gases such as CO_2_ and K_2_O that etch the carbon matrix, thereby forming micropores and mesopores [7]. The use of microwave irradiation accelerates this process by providing rapid and uniform heating, which enhances the evolution of gaseous species and promotes the expansion and interconnection of pores. This mechanism results in a hierarchical pore structure, consistent with the adsorption behavior observed in the BET isotherms, including the hysteresis loop indicative of mesoporous formation.

Table 1 shows the physicochemical properties of the activated carbon samples. As shown in the Table, the total surface area increased markedly, from 25 m^2^/g at 400 °C to 634 m^2^/g at 600 °C. This was accompanied by a progressive increase in total pore volume (from 0.183 cm^3^/g to 0.483 cm^3^/g) and average pore diameter (from 3.05 nm to 28.89 nm). These changes suggest enhanced pore development and structural expansion at higher activation temperatures, likely due to greater volatile release and widening of existing pores. An increase in activation temperature from 400 °C to 500 °C resulted in an enhancement of the specific surface area by a f actor of more than 11, as well as the appearance of micropores. The iodine number, a well-established indicator of microporosity and adsorption capacity for small molecules, also increased significantly with temperature, rising from 385 mg/g at 400 °C to 575 mg/g at 600 °C. It is noteworthy that despite the increase in specific surface area between the CS-A4 and CS-A5 samples, the iodine number decreased by 79 mg/g. This phenomenon may be attributed to a change in the chemistry of the adsorbent surface. However, an increase in temperature to 600 °C resulted in an increase in the iodine number to 575 mg/g. This phenomenon can be attributed to enhanced surface development and the formation of additional oxygen groups. Furthermore, an increase in the activation temperature resulted in an increase in the percentage of ash content. However, as the temperature increased, the yield of the activated carbon decreased, with a range of results from 18.04% to 10.90%. Overall, the results demonstrate that higher activation temperatures substantially improve the textural properties of the activated carbon, particularly favouring mesopore formation. This is advantageous for adsorbing larger molecules, such as methylene blue. CS-A6 exhibited the highest surface area, pore volume and iodine number among the samples, making it the most suitable candidate for adsorption applications.

Comparing the results obtained with the adsorbents described in the literature, it can be concluded that the carbon material obtained from grape seeds by activation with potassium carbonate at 600 °C had a specific surface area of 358 m^2^/g [2]. This value is almost 300 m^2^/g lower than the value obtained for sample CS-A6. The specific surface area of activated carbon obtained from corn stalk by activation with potassium carbonate at 450 and 600 °C was found to be 58 m^2^/g and 542 m^2^/g, respectively [19]. In summary, the activated carbons obtained are characterised by a similar or even superior specific surface area when compared to materials obtained at a similar temperature using conventional heating methods.

Table 2 summarizes the acid–base surface properties of the activated carbon samples (CS-A4, C S-A5, and CS-A6), including the pH of aqueous extracts and the concentrations of acidic and basic oxygen functional groups as determined by the Boehm titration method. Across all samples, a predominance of acidic functional groups was observed, consistent with the low pH values (5.37–5.38) of their aqueous extracts. The data show a clear trend of increasing acidic group concentration with activation temperature: from 1.32 mmol/g for CS-A4 (400 °C), to 1.30 mmol/g for CS-A5 (500 °C), and reaching 1.93 mmol/g for CS-A6 (600 °C). A slight but consistent increase in basic group content was also noted, from 0.43 mmol/g to 0.53 mmol/g across the same temperature range. These results indicate that higher activation temperatures promote the formation of more oxygen-containing functional groups on the activated carbon surface, especially acidic ones. This aligns with previous findings that the oxidative processes at elevated temperatures enhance the development of polar surface functionalities, contributing to the hydrophilic and reactive nature of the adsorbent material [20]. Despite this increase, acidic groups remained dominant, which correlates with the persistently low pH of the activated carbon extracts.

**Figure 2:**
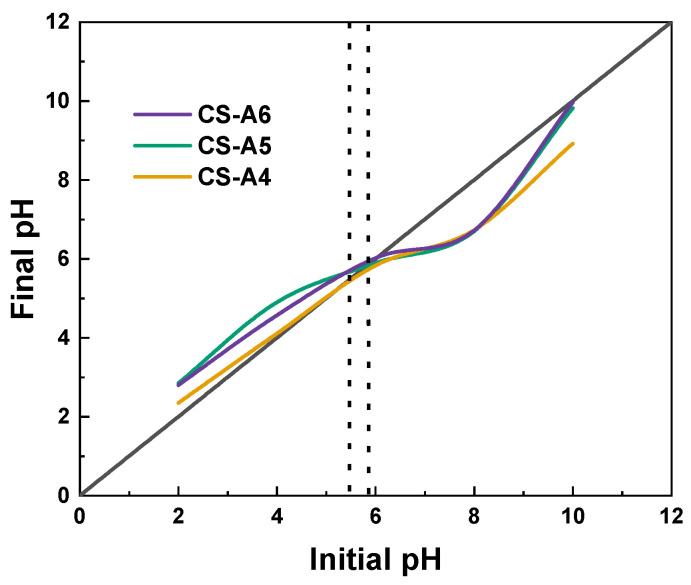
The point of zero charge (pHpzc) of tested activated carbons.

The pHpzc values of CS-A4, CS-A5 and CS-A6 were determined using the drift method, as illustrated in Figure 2. The pHpzc for the obtained samples was approximately 5.5 for the CS-A4 sample and approximately 5.9 for the CS-A5 and CS-A6 samples. It can be concluded that the pHpzc value of the resulting adsorbent increased as the mass ratio of precursor to activator increased. In accordance with the definition of the point of zero charge, the surface of activated carbons is negatively charged when pH > pHpzc and positively charged below this value.

Elemental composition of the samples was determined by both elemental analysis (Table 3) and X-ray Photoelectron Spectroscopy (Table 4). Elemental analysis (Table 3) revealed clear compositional changes across samples CS-A4, CS-A5, and CS-A6. Carbon content decreased progressively from 73.71 wt.% in CS-A4 to 65.33 wt.% in CS-A6, accompanied by a similar decline in hydrogen (4.18 wt.% to 2.69 wt.%). In contrast, oxygen content increased markedly from 17.08 wt.% to 27.01 wt.%, while nitrogen remained relatively constant (~5 wt.%). These trends indicate a gradual oxidation process, characterized by the enrichment of oxygen-containing functional groups and depletion of carbon- and hydrogen-rich structures, reflecting progressive chemical modification.

XPS results (Table 2) reveal clear trends in surface composition with increasing activation temperature. Sample CS-A4, activated at 400 °C, shows a carbon content of 89.27 at.%, oxygen of 9.93 at.%, and nitrogen of 0.81 at.%. Upon increasing the activation temperature to 500 °C (CS-A5), the carbon content rises to 92.60 at.%, while oxygen decreases markedly to 5.45 at.%; nitrogen also increases slightly to 1.96 at.%. At the highest activation temperature of 600 °C (CS-A6), carbon slightly decreases to 89.39 at.%, oxygen rises to 7.17 at.%, and nitrogen further increases to 3.43 at.%. These variations indicate that thermal treatment strongly affects the surface chemistry. The increase in carbon content and decrease in oxygen at 500 °C suggests partial removal of oxygenated functional groups, likely due to thermal decomposition of surface-bound species. However, at 600 °C, the slight drop in carbon and rise in oxygen may indicate re-oxidation of the surface or exposure of oxygen-containing moieties from deeper layers as the structure evolves. Nitrogen content steadily increases with temperature, possibly due to nitrogen species becoming more surface-exposed or more thermally stable compared to oxygen functionalities.

While both techniques confirm that carbon is the dominant element, notable differences in the reported values are observed. Elemental analysis indicates carbon contents of 65–74 wt.%, oxygen levels of 17–27 wt.%, and nitrogen levels of ~5 wt.% for all samples. In contrast, XPS data shows significantly higher carbon contents (~89–93 at.%) and lower oxygen and nitrogen values. These discrepancies arise primarily from the fundamental differences between the two methods. Elemental analysis measures the bulk composition of the entire sample, providing weight percentages of each element. XPS, on the other hand, is a surface-sensitive technique, probing only the top 10 nanometers and reporting atomic percentages. The higher carbon content and lower oxygen/nitrogen levels in XPS likely reflect a carbon-rich surface layer, while oxygen and nitrogen are more prevalent in the bulk or associated with functional groups beneath the immediate surface. Additionally, differences between weight percent (wt.%) and atomic percent (at.%) further contribute to variations, as lighter elements like hydrogen significantly influence wt.% values but are not detected by XPS.

It is important to note that adsorption takes place at the solid–liquid interface, and therefore the surface composition determined by XPS is particularly relevant for understanding dye removal mechanisms. The higher surface carbon content observed by XPS suggests the presence of aromatic domains conducive to π–π stacking with dye molecules, while the oxygen- and nitrogen-containing groups detected at the surface contribute to polar interactions such as hydrogen bonding and electrostatic attraction. In contrast, the higher bulk oxygen and nitrogen contents revealed by elemental analysis likely represent functionalities embedded deeper within the material that are less directly accessible, but may influence pore development and surface reactivity.

**Figure 3:**
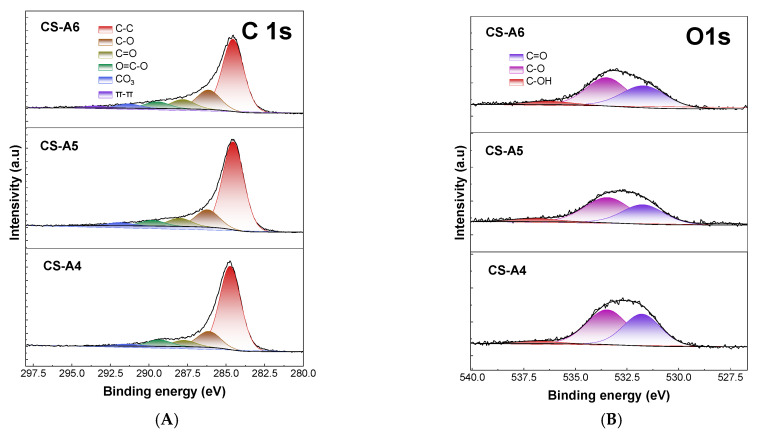
(**A**) The XPS carbon (C1s) spectra of obtained activated carbon samples. (**B**) The XPS oxygen (O1s) spectra of obtained activated carbon samples.

The high-resolution XPS spectra of C1s are shown in Figure 3A and of O1s in Figure 3B. The C1s spectra were found to contain a minimum of five to six resolved peaks, which were subsequently assigned to the following functional groups: C-C, C-O, C=O, O-C=O, CO_3_ and π-π bond [21]. CS-A6 contains the highest amount and diversity of functional groups due to better developed specific surface area. XPS analysis of the C1s spectra reveals the presence of oxygen-containing groups such as C-O, C=O, O-C=O, and CO_3_, which contribute to the adsorption of polar molecules via hydrogen bonding or dipole interactions.

The O1s spectra were deconvoluted into three distinct peaks. The peaks in the range of 531.15–531.7 eV are attributed to oxygen double-bonded to carbon in carbonyl groups (C=O), those at 531.15–531.7 eV correspond to C-O groups, and the peaks around 536.8 eV are associated with hydroxyl groups (C-OH) [21].

### Adsorption

**Figure 4:**
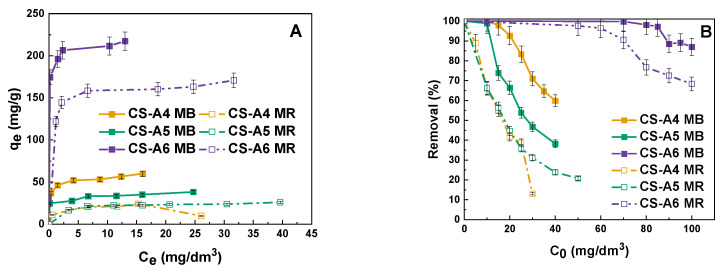
Isotherms (**A**) and correlation between removal (%) of polutant solution and its initial concentration (**B**) of methylene blue (MB) and methyl red (MR) by obtained samples.

As shown in Figure 4, the adsorption isotherms of methylene blue and methyl red are presented. The sorption capacity of the sample was found to depend on the activation temperature. The sample obtained at 600 °C exhibited a markedly higher sorption capacity compared to those prepared at lower temperatures. The maximum sorption capacities for methylene blue were as follows: the CS-A6 sample showed 217 mg/g, while the CS-A5 and CS-A4 samples exhibited 38 mg/g and 60 mg/g, respectively. For methyl red, the corresponding values were 171, 24, and 24 mg/g, respectively.

Upon analysing the curves presented in Figure 4B, it is evident that each of the obtained adsorbents had a distinct range of initial concentrations where a change in % removal is noticeable. Therefore, different initial concentrations were chosen for each sample for further testing. The concentration of methylene blue was 30 mg/dm^3^ for the CS-A4 sample, 20 mg/dm^3^ for the CS-A5 sample and 90 mg/dm^3^ for the CS-A6 sample. The concentration of methyl red was 20 mg/dm^3^ for the CS-A4 and CS-A5 sample and 80 mg/dm^3^ for CS-A6 sample.

A comparison can be drawn between the sorption capacities obtained for methylene blue and the values obtained for other carbon adsorbents. Activated carbon derived from mangosteen peels, when treated with K_2_CO_3_ at 700 °C for 120 min, exhibited a sorption capacity of 86 mg/g for MB [22]. Macadamia nutshell-based activated carbon demonstrated a significantly higher capacity of 250 mg/g for MB under similar chemical activation with K_2_CO_3_ at 650 °C for 60 min [23]. For comparison, the CS-A6 sample in this study, prepared at 600 °C, exhibited a sorption capacity of 217 mg/g, which is comparable to macadamia nutshell-based carbon and considerably higher than that of mangosteen peel-derived carbon. These results indicate that the activation conditions and precursor type strongly influence adsorption performance, with CS-A6 showing competitive efficiency among the tested materials.

**Figure 5:**
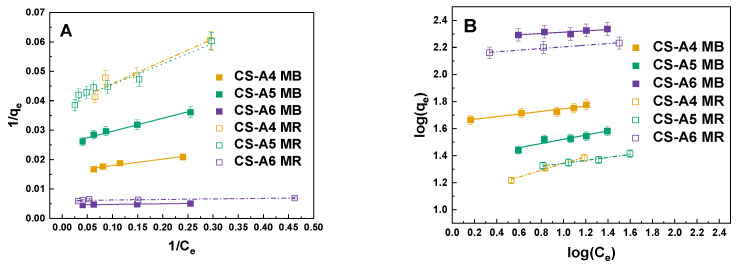
Linear fitting for MB and MR on obtained activated carbon to (**A**) Langmuir model and (**B**) Freundlich model.

The adsorption behaviors of methylene blue (MB) and methyl red (MR) onto the CS-A series activated carbons were investigated using Langmuir and Freundlich isotherm models (Figure 5), with the corresponding fitting parameters summarized in Table 5 and Table 6. For both dyes, the experimental adsorption capacity (q_exp_) increased significantly with activation temperature, reflecting the enhanced sorption performance of samples with higher surface area and porosity. Specifically, MB adsorption increased from 38 mg/g for CS-A5 to 217 mg/g for CS-A6, while MR adsorption rose from 24 mg/g for CS-A4 to 171 mg/g for CS-A6.

The Langmuir model provided an excellent fit for MB adsorption across all samples (R^2^ > 0.95), with CS-A6 showing a maximum monolayer capacity (qₘ) of 220 mg/g, closely matching the experimental value (217 mg/g). This indicates that MB adsorption predominantly occurs as monolayer coverage on relatively homogeneous surfaces [24]. Conversely, the Freundlich model showed weaker correlations for MB, particularly for CS-A6 (R^2^ = 0.683), suggesting limited multilayer adsorption. In contrast, MR adsorption demonstrated a different trend. While the Langmuir model adequately described adsorption on CS-A4 and CS-A5 (R^2^ > 0.90), it exhibited a poor fit for CS-A6 (R^2^ = 0.673), indicating that the assumptions of uniform monolayer adsorption are not valid for this material. The high activation temperature of CS-A6 produces a more heterogeneous surface, enriched with diverse oxygen- and nitrogen-containing functionalities and a wider pore distribution, generating sites of varying adsorption energy. This heterogeneity favors multilayer adsorption and complex interactions, consistent with the excellent fit of the Freundlich model (R^2^ > 0.97), with CS-A6 exhibiting the highest K_F_ value (139.07 mg/g (dm^3^/mg)^1^/ⁿ). These findings indicate that MR uptake on CS-A6 is predominantly governed by adsorption on heterogeneous surfaces rather than conventional monolayer coverage [25].

The adsorption mechanism of the caraway seed-derived activated carbons can be explained by the interplay of pore structure and surface chemistry. The BET analysis shows that the materials possess hierarchical porosity, with micropores providing high surface area for adsorption of small molecules, and mesopores facilitating diffusion and adsorption of larger dyes such as methylene blue. XPS analysis of the C1s spectra indicates the presence of oxygen- and nitrogen-containing functional groups, including C-O, C=O, O-C=O, and CO_3_, which can interact with polar molecules through hydrogen bonding, dipole interactions, or electrostatic attraction. Additionally, aromatic C-C/C-H domains support π-π interactions with aromatic dyes. The combination of well-developed pore networks and surface functionalities explains the high adsorption capacities observed and the differences in behavior between methylene blue and methyl red across the sample.

**Figure 6:**
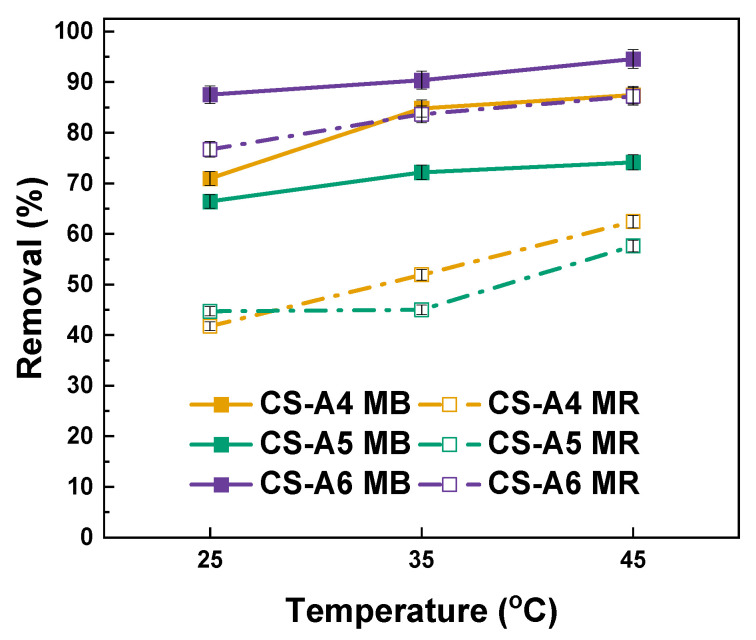
Effect of temperature of the aqueous solution of dye on removal.

Thermodynamic analysis offers critical insight into whether adsorption occurs predominantly via physisorption or chemisorption. Increasing temperature enhances the kinetic energy of dye molecules, promoting their interaction with the adsorbent surface. To assess the thermodynamic behavior, adsorption experiments were conducted at 25, 35, and 45 °C. As shown in Figure 6 adsorption capacity slightly increased with temperature. From an economic perspective, the process is advantageous, as high performance is achieved at room temperature without the need for additional energy input. The process is effective in a room temperature.

Thermodynamic parameters for methylene blue (MB) and methyl red (MR) adsorption on the activated carbon samples are summarized in Table 7 and Table 8. Across all samples and both dyes, the negative values of Gibbs free energy (*ΔG*^0^) confirm that adsorption is a spontaneous process. Moreover, the magnitude of *ΔG*^0^ becomes more negative with increasing temperature, indicating that higher temperatures favor adsorption, consistent with an endothermic nature of the process [26]. For MB adsorption (Table 7), CS-A6 shows the most negative *ΔG*^0^ values (−7.10 to −9.99 kJ/mol) compared to CS-A4 and CS-A5, indicating stronger spontaneity and higher affinity for MB molecules. Enthalpy (*ΔH*^0^) values are positive for all samples (14.71–41.52 kJ/mol), confirming the endothermic character of the adsorption. The relatively high ΔH° and entropy (*ΔS*^0^) values for CS-A6 (35.78 kJ/mol and 143.41 J/mol·K, respectively) suggest enhanced structural reorganization and increased randomness at the solid-liquid interface during adsorption [27].

For MR adsorption (Table 8), a similar trend is observed. CS-A6 again exhibits the most negative *Δ*G^0^ values (−5.22 to −7.49 kJ/mol), highlighting its superior adsorption performance compared to CS-A4 and CS-A5. Positive *Δ*H^0^ values (20.27–33.07 kJ/mol) indicate an endothermic process, while the relatively high *Δ*S^0^ values suggest increased disorder at the adsorbent–adsorbate interface, particularly for CS-A6 (113.99 J/mol×K) [28]. Overall, CS-A6 demonstrates the most favorable thermodynamic parameters for both dyes, reflecting the beneficial effect of higher activation temperature (600 °C) in enhancing surface characteristics and adsorption performance.

**Figure 7:**
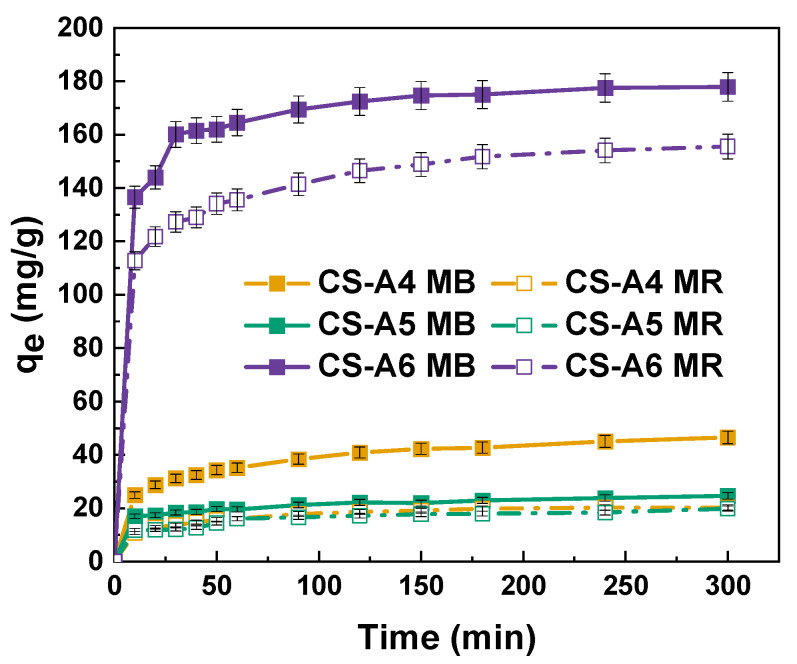
Effect of contact time on the sorption capacities of the activated carbon samples.

**Figure 8:**
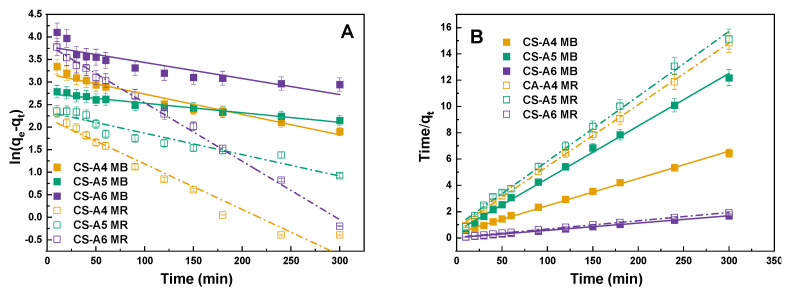
Linear fitting for methylene blue on obtained activated carbon to (**A**) pseudo-first-order model and (**B**) pseudo-second-order model.

The effect of contact time on the dye removal efficiency of the activated carbons was investigated to determine the period required to achieve adsorption equilibrium. As illustrated in Figure 7, equilibrium was reached within approximately 100 min, indicating the rapid adsorption kinetics of the materials. Such short equilibrium times are advantageous for practical applications, as they minimize operational duration and energy consumption. The resulting graphs for pseudo-first-order and pseudo-second-order are shown in Figure 8. Kinetic modeling results for methylene blue and methyl red adsorption are presented in Table 9 and Table 10.

For MB adsorption (Table 9), the pseudo-second-order model provides a better fit than the pseudo-first-order model, as indicated by higher correlation coefficients (R^2^ = 0.997–0.999) and closer agreement between experimental and calculated q_e_ values. In contrast, the pseudo-first-order model shows lower R^2^ values (0.781–0.966) and significant deviations in q_e_, suggesting it does not adequately describe the adsorption mechanism. Similarly, MR adsorption (Table 10) is also best represented by the pseudo-second-order model, with R^2^ values of 0.999 for all samples and close agreement between calculated and experimental q_e_. The pseudo-first-order model, while yielding reasonably high R^2^ values (0.926–0.996), underestimates q_e_, particularly for CS-A6. These results indicate that adsorption of both dyes onto the activated carbons is primarily governed by chemisorption involving electron sharing or exchange between dye molecules and active sites on the adsorbent surface [29]. The notably higher rate constants (k_2_) for CS-A6 further confirm its superior adsorption kinetics, attributed to enhanced surface properties resulting from activation at 600 °C.

**Figure 9:**
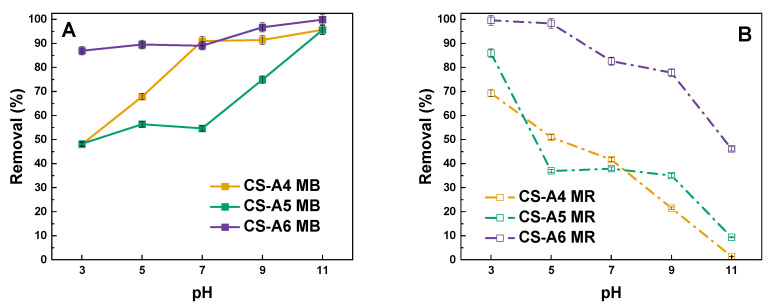
Effect of pH of the aqueous solution of (**A**) methylene blue and (**B**) methyl red on removal (adsorbent mass: 20 mg, volume of dye solution: 0.05 dm^3^).

The sorption capacities of activated carbons have been shown to be susceptible to the presence of hydrogen and hydroxyl ions in aqueous solutions. These ions have been demonstrated to alter the charge on the surface of the adsorbent. The effect of pH on the removal process is illustrated in Figure 9.

Analysis of the graph indicates that increasing pH levels enhances the sorption capacity for the cationic dye methylene blue. In contrast, methyl red shows a pronounced decline in removal efficiency as pH rises. This behavior can be attributed to the adsorbent surface being positively charged under acidic conditions, which promotes electrostatic attraction between the activated carbon and anionic dye molecules such as MR, thereby improving adsorption. At alkaline pH, however, the abundance of hydroxyl ions likely competes with MR molecules for adsorption sites, hindering the process. Interestingly, the opposite trend was observed for methylene blue. Similar pH-dependent adsorption patterns have been reported in previous studies [30,31].

The determination of the point of zero charge (pHpzc) further supports these observations. Based on the pH drift method applied to the adsorption data, the pHpzc of the activated carbon was found to lie between 5.5 and 5.9, depending on the dataset. This means that at pH values below the pHpzc, the adsorbent surface is predominantly positively charged, favoring the adsorption of anionic dyes such as methyl red. Conversely, at pH values above the pHpzc, the surface becomes negatively charged, thereby enhancing the removal of cationic dyes such as methylene blue.

## 3. Materials and Methods

### 3.1. Precursor and Activated Carbon Samples Preparation

Caraway seeds (CS), which failed to meet quality control standards and were thus classified as waste from the herbal industry, were used as precursors for activated carbon production. The raw material was impregnated with potassium carbonate in a precursor-to-activator ratio of 1:2. The impregnated samples were then carbonized and activated at temperatures of 400 °C (CS-A4), 500 °C (CS-A5), and 600 °C (CS-A6) for 30 minuts in a nitrogen atmosphere with a flow rate of 270 cm^3^/min. All thermal processes were conducted in a microwave oven (Phoenix, CEM Corporation, Matthews, IL, USA) with a heating rate of 10 °C/min. After activation, the resulting activated carbon samples were washed with hydrochloric acid and rinsed with boiling distilled water. The materials were then dried to obtain a stable solid form. The adsorption capacities of the activated carbons were assessed using aqueous solutions of methylene blue and methyl red. Both analytical-grade dyes were sourced from Merck (Darmstadt, Germany), while all other chemicals used in the study were obtained from Sigma-Aldrich (Burlington, MA, USA), also of analytical grade.

### 3.2. Characterization of Resulted Activated Carbon Samples

The textural properties of the prepared samples were evaluated using nitrogen adsorption–desorption isotherm measurements carried out at −196 °C with an AutosorbiQ analyzer (Quantachrome Instruments, Boynton Beach, FL, USA).

The iodine number is measured following the guidelines of the ASTM D4607-94 method.

The presence of surface oxygen-containing functional groups with acidic and basic characteristics in the resulting activated carbon samples was assessed using the Boehm titration method (Boehm, 1994) [32,33].

The methodology for determining the point of zero charge (pHpzc) for activated carbon was assessed using the method described in the previous study [30].

X-ray photoelectron spectroscopy (XPS) analysis was performed using an ultra-high vacuum photoelectron spectrometer equipped with a Phoibos150 NAP analyzer (Specs, Berlin, Germany). The measurements were conducted as described in the previous article [30].

Elemental analysis was performed on Elemental Analyzer Vario EL III (Elementar, Hesse, Germany).

### 3.3. Adsorption Studies

Methylene blue and methyl red were selected as the model dyes for this study. Aliquots of 20 mg from each activated carbon sample were added to 0.05 dm^3^ of aqueous dye solution, with initial dye concentrations ranging from 10 to 100 mg/dm^3^. The mixtures were agitated at room temperature (22 ± 1 °C) using a Heidolph laboratory shaker set to 300 rpm for a duration of 24 h. Following adsorption, the absorbance of each solution was measured spectrophotometrically at a wavelength of 665 nm for methylene blue and of 443 using a dual-beam UV–VIS spectrophotometer (Cary Bio 100 model, Varian).

The adsorption capacity of each activated carbon sample at equilibrium (qₑ, mg/g) was calculated using the following Equation (1):

(1) $$q_{e}=\frac{\left(C_{0-}C_{e}\right)}{m}\times V$$

where *C_0_* and *C_e_*—the initial and equilibrium concentrations (mg/dm^3^) of the dye in solution; respectively; *m*—the mass of the activated carbon (g); *V*—the volume of the solution (dm^3^).

To identify the most appropriate model describing dye adsorption onto activated carbon, the linear forms of the Langmuir and Freundlich isotherm equations were applied. The Langmuir isotherm is expressed in its linear form as follows (2):

(2) $$\frac{1}{q_{e}}=\frac{1}{q_{max}}+\frac{1}{K_{L}q_{m}}\times \frac{1}{C_{e}}$$

where *q_e_*—the equilibrium amount of adsorbed dye (mg/g); *K_L_*—Langmuir equilibrium constant (dm^3^/mg); a *q_max_*—the maximum adsorption capacity of the adsorbent (mg/g).

The Freundlich isotherm can be represented by the following linear Equation (3):

(3) $${logq}_{e}={logK}_{F}+\frac{1}{n}{logC}_{e}$$

where *K_F_*—Freundlich equilibrium constant (mg/g(dm^3^/mg)^1/*n*^); 1/*n*—the adsorption intensity constant.

For further research the initial concentrations for each adsorbent were selected based on their different adsorption capacities. The concentration of methylene blue was 30 mg/dm^3^ for the CS-A4 sample, 20 mg/dm^3^ for the CS-A5 sample and 90 mg/dm^3^ for the CS-A6 sample The concentration of methyl red was 20 mg/dm^3^ for the CS-A4 and CS-A5 sample and 80 mg/dm^3^ for CS-A6 sample.

In addition, the study examined the effect of process temperature on the adsorption of aqueous dyes solutions. To evaluate the influence of temperature on adsorption efficiency, triplicate samples (20 mg each) of each activated carbon was prepared and treated analogously to the initial adsorption tests. The samples were immersed in dye solutions and shaken for 24 h at controlled temperatures of 25 °C, 35 °C, and 45 °C. After the adsorption period, the mixtures were centrifuged using a laboratory centrifuge, and the supernatants were analyzed by spectrophotometry.

Thermodynamic parameters were then calculated using the following Equations (4)–(6):

(4) $$∆G^{0}=-RTlnK_{d}$$

(5) $$∆G^{0}=∆H^{0}-T∆S^{0}$$

(6) $$lnK_{d}=\frac{∆S^{0}}{R}+\frac{∆H^{0}}{RT}$$

where: *ΔG^0^*—Gibbs free energy; *R*—universal constant (8.314 J/mol × K); *T*—temperature (K); *ΔH^0^*—enthalpy change; *Δ*S^0^—entropy change; *K_d_*—thermodynamic equilibrium constant.

To characterize the adsorption kinetics of methylene blue and methyl red on the activated carbon samples, four kinetic models were applied: the pseudo-first-order model (7) and the pseudo-second-order model (8):

(7) $$\log\left(q_{e}-q_{t}\right)=logq_{e}-\frac{k_{1}}{2.303}t$$

(8) $$\frac{t}{q_{t}}=\frac{1}{k_{2}q_{e^{2}}}+\frac{t}{q_{e}}$$

where *q_e_*—the equilibrium amount of adsorbed dye (mg/g); *q_t_*—the amount of adsorbed dye over time (mg/g); *t*—the process time (min); *k*_1_—the pseudo-first-order adsorption constant (1/min); *k*_2_—the pseudo-second-order adsorption constant (g/mg × min).

## 4. Conclusions

The findings suggest that caraway seeds can be effectively utilised as precursors for the fabrication of biocarbon adsorbents through the process of microwave-assisted chemical activation with K_2_CO_3_. This approach has been demonstrated to be efficacious in the removal of methylene blue and methyl red from aqueous solutions. The physicochemical characterisation of the materials obtained revealed that their specific surface area ranged from 25 to 634 m^2^/g, while their iodine number varied between 306 and 575 mg/g. XPS analysis of the C1s spectra reveals the presence of oxygen-containing groups, including C-O, C=O, O-C=O and CO_3_, which contribute to the adsorption of polar molecules by hydrogen bonding or dipole interactions. The spontaneous, endothermic process of adsorption of MR and MB onto the obtained sorbents is driven by increased entropy. The findings of this study lend support to the hypothesis that microwave-activated activated carbon, derived from agricultural waste, has the potential to function as an efficient and sustainable adsorbent for the removal of dyes from aqueous systems. The kinetic analysis confirms that the adsorption of MR and MB onto the obtained carbon materials is best described by the pseudo-second-order model. In the course of the present study, a range of materials were subjected to rigorous testing. Among these materials, CS-A6 sample demonstrated the highest level of efficacy, indicating its strong potential as a low-cost and efficient adsorbent for dye removal applications.

The subsequent phase of the research will concentrate on the modification of activation parameters, encompassing gas flow rate and activation time, with the objective of minimising overall process costs. Furthermore, it is anticipated that elevated activation temperatures will lead to a pronounced enhancement in the properties of the resultant carbons. This aspect will be addressed in forthcoming research. Subsequent research will comprise an examination of desorption processes, in conjunction with an assessment of the sorption capacity of the adsorbents in wastewater.

The practical applicability of the obtained adsorbents is promising. Caraway seeds represent a low-cost and abundant precursor, while microwave-assisted activation with K_2_CO_3_ is an energy-efficient process suitable for upscaling. While further investigation in the form of regeneration and desorption studies is required to fully ascertain the potential of these carbons, the structural stability of the prepared carbons indicates good potential for reusability. The aforementioned factors, when considered in conjunction with the demonstrated adsorption efficiency, lend support to the feasibility of implementing the proposed adsorbents within real wastewater treatment systems.

## Figures and Tables

**Table 1:** Textural parameters, iodine number and ash content of obtained activated carbon samples.

Sample	Surface Area ^1^ (m^2^/g)		Pore Volume (cm^3^/g)		Average Pore Size (nm)	Iodine Number (mg/g)	Ash Content (%)
	Total	Microporous	Total	Microporous			
CS-A4	25	0	0.183	0	28.89	385	1.31
CS-A5	284	149	0.278	0.083	3.92	306	2.11
CS-A6	634	406	0.483	0.235	3.05	575	3.41

^1^ error range 2–5%.

**Table 2:** Acid-base properties of the obtained activated carbon samples.

Sample	pH	Acidic Oxygen Functional Groups (mmol/g)	Basic Oxygen Functional Groups (mmol/g)
CS-A4	5.37 ± 0.01	1.32 ± 0.07	0.43± 0.02
CS-A5	5.37 ± 0.01	1.30 ± 0.07	0.43 ± 0.02
CS-A6	5.38 ± 0.01	1.93± 0.10	0.53 ± 0.03

**Table 3:** Elemental analysis of the obtained activated carbon samples (wt.%).

Sample	C wt.%	N wt.%	H wt.%	O wt.% *
CS-A4	73.71	5.03	4.18	17.08
CS-A5	72.16	4.95	2.81	20.08
CS-A6	65.33	4.97	2.69	27.01

*—By difference; method error ≤0.3%.

**Table 4:** Relative contents of elements (at.%) for obtained samples based on XPS analysis.

Sample	C at.%	O at.%	N at.%
CS-A4	89.27	9.93	0.81
CS-A5	92.60	5.45	1.96
CS-A6	89.39	7.17	3.43

**Table 5:** The values of constants determined for the linear Langmuir and Freundlich models for experimental data of MB.

Isotherm	Parameters	Sample		
		CS-A4	CS-A5	CS-A6
	q_exp_ (mg/g)	60	38	217
Langmuir	K_L_ (dm^3^/mg)	0.715	0.589	2.121
	q_m_ (mg/g)	63	39	220
	R^2^	0.951	0.973	0.972
	Adj^2^	0.927	0.964	0.957
Freundlich	K_F_ (mg/g(dm^3^/mg)^1/n^)	44.641	23.240	183.997
	1/n	0.097	0.155	0.048
	R^2^	0.945	0.900	0.6831
	Adj^2^	0.926	0.866	0.578

**Table 6:** The values of constants determined for the linear Langmuir and Freundlich models for experimental data of MR.

Isotherm	Parameters	Sample		
		CS-A4	CS-A5	CS-A6
	q_exp_ (mg/g)	24	26	171
Langmuir	K_L_ (dm^3^/mg)	0.501	0.442	3.201
	q_m_ (mg/g)	26	26	165.289
	R^2^	0.909	0.933	0.673
	Adj^2^	0.861	0.920	0.564
Freundlich	K_F_ (mg/g(dm^3^/mg)^1/n^)	12.320	17.117	139.072
	1/n	0.252	0.0110	0.061
	R^2^	0.984	0.970	0.977
	Adj^2^	0.968	0.953	0.955

**Table 7:** Thermodynamic parameters of methylene blue adsorption on the obtained activated carbon.

Sample	Temperature (°C)	*∆G*^0^ (kJ/mol)	*∆H*^0^ (kJ/mol)	*∆S*^0^ (J/mol × K)
CS-A4	25	−4.48	41.52	155.15
	35	−6.75		
	45	−7.55		
CS-A5	25	−3.96	14.71	62.86
	35	−4.94		
	45	−5.05		
CS-A6	25	−7.10	35.78	143.41
	35	−8.09		
	45	−9.99		

**Table 8:** Thermodynamic parameters of methyl red adsorption on the obtained activated carbon.

Sample	Temperature (°C)	*∆G*^0^ (kJ/mol)	*∆H*^0^ (kJ/mol)	*∆S*^0^ (J/mol × K)
CS-A4	25	−1.45	33.07	115.78
	35	−2.54		
	45	−3.77		
CS-A5	25	−1.74	20.27	73.18
	35	−1.83		
	45	−3.24		
CS-A6	25	−5.22	28.69	113.99
	35	−6.53		
	45	−7.49		

**Table 9:** Kinetic models parameters for methylene blue adsorption.

Kinetics Model	Parameters	Sample		
		CS-A4	CS-A5	CS-A6
	q_e_ (mg/g)	53	33	197
Pseudo-first-order	k_1_ (1/min)	1.51 × 10^−5^	7.07 × 10^−6^	1.19 × 10^−5^
	R^2^	0.966	0.952	0.781
	q_e/cal_ (mg/g)	24	16	44
Pseudo-second-order	k_2_ (g/mg × min)	2.31 × 10^−3^	3.26 × 10^−3^	8.61 × 10^−3^
	R^2^	0.997	0.997	0.999
	q_e/cal_ (mg/g)	50	31	198

**Table 10:** Kinetic models parameters for methyl red adsorption.

Kinetics Model	Parameters	Sample		
		CS-A4	CS-A5	CS-A6
	q_e_ (mg/g)	21	22	156
Pseudo-first-order	k_1_ (1/min)	3.36 × 10^−5^	1.58 × 10^−5^	4.32 × 10^−5^
	R^2^	0.956	0.926	0.996
	q_e/cal_ (mg/g)	9	10	46
Pseudo-second-order	k_2_ (g/mg × min)	2.62 × 10^−3^	2.30 × 10^−3^	5.11 × 10^−3^
	R^2^	0.999	0.999	0.999
	q_e/cal_ (mg/g)	21	20	159

## Data Availability

Data is contained within the article.
